# NUB1 Regulates Muscle Atrophy in Lung Cancer Cachexia via NF‐κB Signaling Pathway

**DOI:** 10.1002/advs.78492

**Published:** 2026-10-31

**Authors:** Yuqi Han, Yaru Xia, Weiquan Li, Jiaming Wu, Tiexi Yu, Diaoyi Tan, Daojia Miao, Wen Li, Xuejiao Dong, Jiemin Li, Yu Jiao, Guixiao Huang, Xiaoping Zhang, Hongmei Yang

**Affiliations:** ^1^ Department of Pathogenic Biology School of Basic Medicine Tongji Medical College Huazhong University of Science and Technology Wuhan China; ^2^ Department of Urology Union Hospital Tongji Medical College Huazhong University of Science and Technology Wuhan China; ^3^ Institute of Urology Tongji Medical College Huazhong University of Science and Technology Wuhan China; ^4^ Department of Urology The Third Affiliated Hospital of Shenzhen University: Shenzhen Luohu Hospital Group Luohu People's Hospital Shenzhen China; ^5^ Shenzhen Huazhong University of Science and Technology Research Institute Shenzhen China

**Keywords:** cancer‐associated cachexia, muscle atrophy, NF‐κB signaling, NUB1

## Abstract

Cancer‐associated cachexia (CAC) is a devastating multifactorial syndrome characterized by progressive skeletal muscle atrophy. Its mechanisms remain incompletely clarified, with limited effective therapeutic targets. NUB1, a negative regulator of ubiquitin‐like proteins mediating protein degradation, has not been fully elucidated in CAC‐induced muscle atrophy. Here, NUB1 was significantly upregulated in skeletal muscles of Lewis lung carcinoma (LLC) cachexia mice and C2C12 myotube atrophy models stimulated with LLC tumor‐conditioned medium (LCM). Silencing NUB1 alleviated LCM‐induced myotube atrophy *in vitro* and enhanced muscle mass, fiber cross‐sectional area and function in LLC cachectic mice *in vivo*. Mechanistically, upregulated NUB1 recruited IκBα to bind proteasomal subunit S5a, promoting IκBα degradation, thus increasing NF‐κB nuclear translocation and transcription of *Atrogin1* and *MuRF1*. Silencing NUB1 inhibited NF‐κB activation, while TNFα reversed its protective effect. These findings identified NUB1 as a novel driver of CAC‐related muscle atrophy and highlighted the NUB1/IκBα/NF‐κB axis as a promising therapeutic target.

## Abstract 2

NUB1 mediates skeletal muscle atrophy in lung cancer cachexia. Upregulated NUB1 recruits IκBα and S5a, triggering IκBα degradation and NF‑κB nuclear translocation. Activated NF‑κB elevates Atrogin1 and MuRF1, accelerating muscle protein breakdown and leading to progressive atrophy. These findings highlight NUB1 as a promising therapeutic target for mitigating cachexia‐induced muscle wasting.

## 1 Introduction

Cancer‐associated cachexia (CAC) is a progressive systemic metabolic disorder [1], accounts for 30% of all cancer‐related deaths and affects up to 80% of advanced non‐small‐cell lung cancer patients [2, 3]. Skeletal muscle loss, which leads to functional impairment, is one of the most typical features of CAC. In clinical treatment, sufficient nutritional support and current clinical strategies fail to reverse muscle loss in these patients [4, 5, 6]. Thus, there is an urgent need to identify specific molecular targets that control skeletal muscle protein homeostasis [7].

From a pathophysiological perspective, muscle atrophy results from a disrupted balance between myofibrillar protein synthesis and degradation [8]. Numerous studies, including our previous work, have shown that activation of catabolic pathways—particularly the ubiquitin‐proteasome system (UPS) [9, 10] – drives excessive protein breakdown and plays a vital role in this imbalance [11, 12, 13].

Among catabolic signaling networks, the nuclear factor‐κB (NF‐κB) pathway acts as a master regulator of CAC‐associated muscle wasting [14]. Under the canonical activation pathway, tumor necrosis factor α (TNFα) triggers phosphorylation and subsequent proteasomal degradation of NF‑kappa‑B inhibitor alpha (IκBα), thereby releasing NF‐κB for nuclear translocation [15]. Upon activation, NF‐κB transcriptionally upregulates the muscle‐specific E3 ubiquitin ligases Atrogin1 and MuRF1, accelerating ubiquitin‐mediated degradation of myosin heavy chain (MHC) and causing irreversible muscle loss [14].

Notably, this NF‐κB‐mediated muscle protein degradation depends on the ubiquitin‐proteasome system (UPS) [16, 17]. Besides the classical UPS pathway, several ubiquitin‐like (UBL) proteins (including NEDD8, FAT10, and SUMO) and ubiquitin‐like‐ubiquitin‐associated (UBL‐UBA) family members have been recently identified [18, 19, 20]. These proteins are involved in regulating proteasomal substrate recognition and degradation. However, these factors are seldom explored in cancer cachexia‐induced muscle wasting.

Negative regulator of ubiquitin‐like proteins 1 (NUB1) is a UBL‑UBA family member bearing N‑terminal UBL and C‑terminal UBA domains [21, 22]. As an adaptor, NUB1 can directly interact with target proteins and guide them for proteasomal degradation [23], or deliver UBL‑conjugated substrates (such as those undergoing NEDDylation or FAT10ylation) to the 26S proteasome, ultimately regulating protein turnover [24, 25]. NUB1 acts as a tumor suppressor in multiple cancers, including hepatocellular carcinoma [26], gastric cancer and breast cancer [27, 28, 29]. It regulates key cellular processes such as cell cycle progression and apoptosis [27, 30]. In addition, NUB1 has been extensively implicated in protein degradation control in neurodegenerative disorders such as Huntington's disease [31]. However, its expression and functional role in CAC‑related muscle wasting remain completely unknown.

Integrative analysis of GEO cachexia datasets and Reactome degradation gene screening reveal significant NUB1 upregulation in LLC‐induced cachectic muscle and LCM‐treated myotubes. Here, we demonstrate that NUB1 drives cancer cachexia‐associated muscle atrophy by recruiting IκBα to the proteasomal subunit S5a, promoting its degradation and subsequent NF‐κB activation, which upregulates *Atrogin1* and *MuRF1*. Silencing NUB1 alleviates this process. Collectively, these findings uncover a previously unrecognized regulatory mechanism in cancer cachexia and suggest that modulation of the NUB1/S5a/IκBα/NF‐κB axis represents a promising therapeutic strategy for clinical intervention.

## 2 Results

### 2.1 NUB1 is Highly Expressed in Muscle Atrophy Models

A mouse model of lung cancer cachexia was established as previously described (Figure S1) [32, 33]. To identify key regulators of muscle protein metabolism in cancer cachexia, we analyzed four public GEO datasets of cachectic skeletal muscle (NSCLC‐GSE107470 [34, 35], pancreatic cancer KIC‐GSE226898 [36], Lewis lung cancer LLC‐GSE144567 [37], and colon cancer C26‐GSE251864 [38]) and intersected them with protein metabolism‐related genes from the Reactome database. Four candidate genes (*Fbxo32, Thy1, Daxx, Nub1*) were identified (Figure 1A). Among these genes, *Nub1* was significantly upregulated in cachectic skeletal muscle, whereas *Thy1* was downregulated and *Daxx* showed no obvious change (Figure 1B, C; Figure S2A). Consistently, *Nub1* was also markedly increased in C2C12 myotubes treated with LLC‐conditioned medium (LCM), while *Thy1* and *Daxx* levels remained unchanged (Figure 1H; Figure S2B). Unlike *Fbxo32* (*Atrogin1*), *Thy1*, and *Daxx*, which have well‑known roles in muscle related diseases [39, 40, 41, 42], *Nub1* has only been implicated in protein metabolism, prompting our investigation into its potential role in muscle atrophy.

**FIGURE 1:**
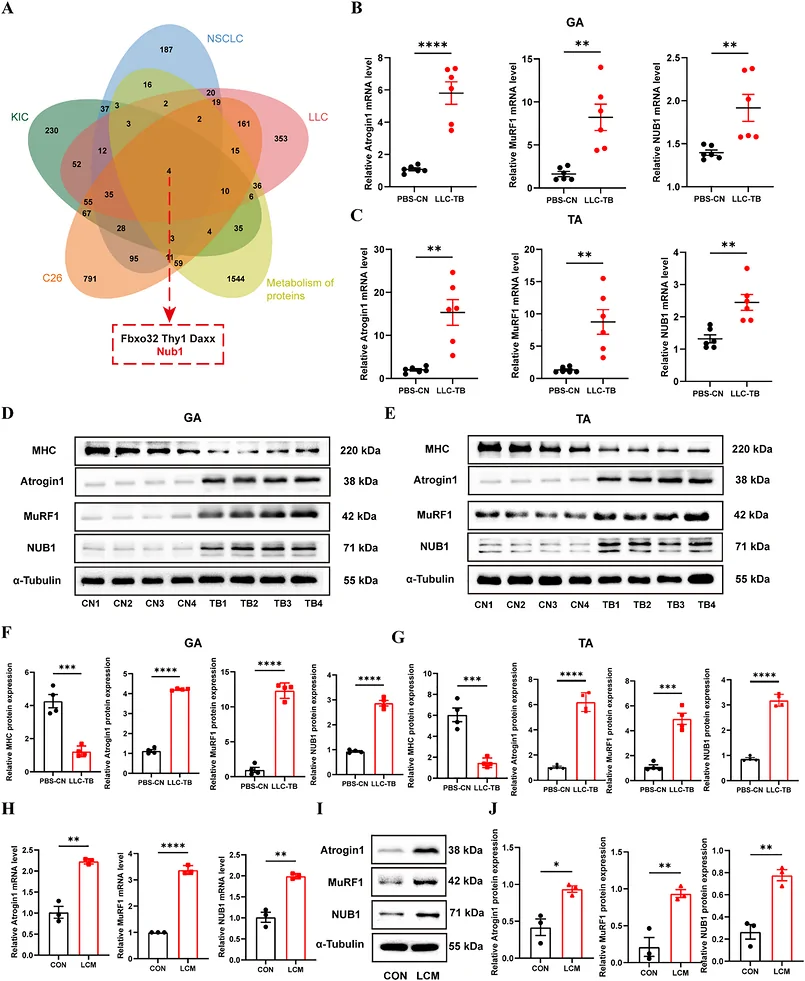
NUB1 is highly expressed in lung cancer cachexia‐induced muscle atrophy models. (A) Venn diagram showing overlapping genes between differentially expressed genes in skeletal muscle from four cachexia models (GEO) and protein metabolism‑related genes (Reactome) (fold change > 1.5, *p* < 0.005). (B, C) qPCR analysis of *Atrogin1*, *MuRF1*, and *Nub1* mRNA levels in gastrocnemius (GA) and tibialis anterior (TA) muscles from control (PBS‑CN) and LLC tumor‑bearing (LLC‑TB) mice (n = 6). (D–G) Western blot analysis of MHC, Atrogin1, MuRF1, and NUB1 protein levels in GA and TA muscles (n = 4). (H) qPCR analysis of *Atrogin1*, *MuRF1*, and *Nub1* mRNA in control (CON) and LLC conditioned medium (LCM)‑treated C2C12 myotubes (n = 3). (I, J) Western blot analysis of Atrogin1, MuRF1, and NUB1 proteins in CON and LCM groups. Data are mean ± SEM from ≥3 independent biological replicates (n = number of independent experiments). Two‐group comparisons used two‐tailed Student's t‐test; multiple groups were compared by one‐way ANOVA with appropriate post‐hoc test. *p < 0.05, **p < 0.01, ***p < 0.001, ****p < 0.0001; ns, not significant.

At the protein level, Atrogin1 and MuRF1 were upregulated and MHC was downregulated in gastrocnemius (GA) and tibialis anterior (TA) muscles of tumor‑bearing mice, confirming severe muscle atrophy. Meanwhile, NUB1 protein was significantly increased in the tumor‑bearing (TB) group (Figure 1D–G). Similarly, NUB1 protein was strongly elevated in LCM‑treated myotubes (Figure 1I,J). These results demonstrated that NUB1 was upregulated in both *in vivo* and *in vitro* models of lung cancer cachexia‑induced muscle atrophy, suggesting it may be a key regulator in this process.

### 2.2 Silencing NUB1 Alleviates Myotube Atrophy in Lung Cancer Cachexia

*In vitro*, LCM treatment induced typical myotube atrophy, characterized by increased NUB1, Atrogin1, and MuRF1 expression and decreased MHC expression. (Figure 2A; Figure S3A). Immunostaining for MHC confirmed that LCM markedly reduced myotube diameter and length (Figure 2B,C). In C2C12 myotubes, lentivirus‑mediated NUB1 knockdown (shNUB1) effectively reduced NUB1 expression, lowered Atrogin1 and MuRF1 levels, and restored MHC expression (Figure 2D; Figure S3B). Morphologically, NUB1 silencing promoted myotube growth and size (Figure 2E,F).

**FIGURE 2:**
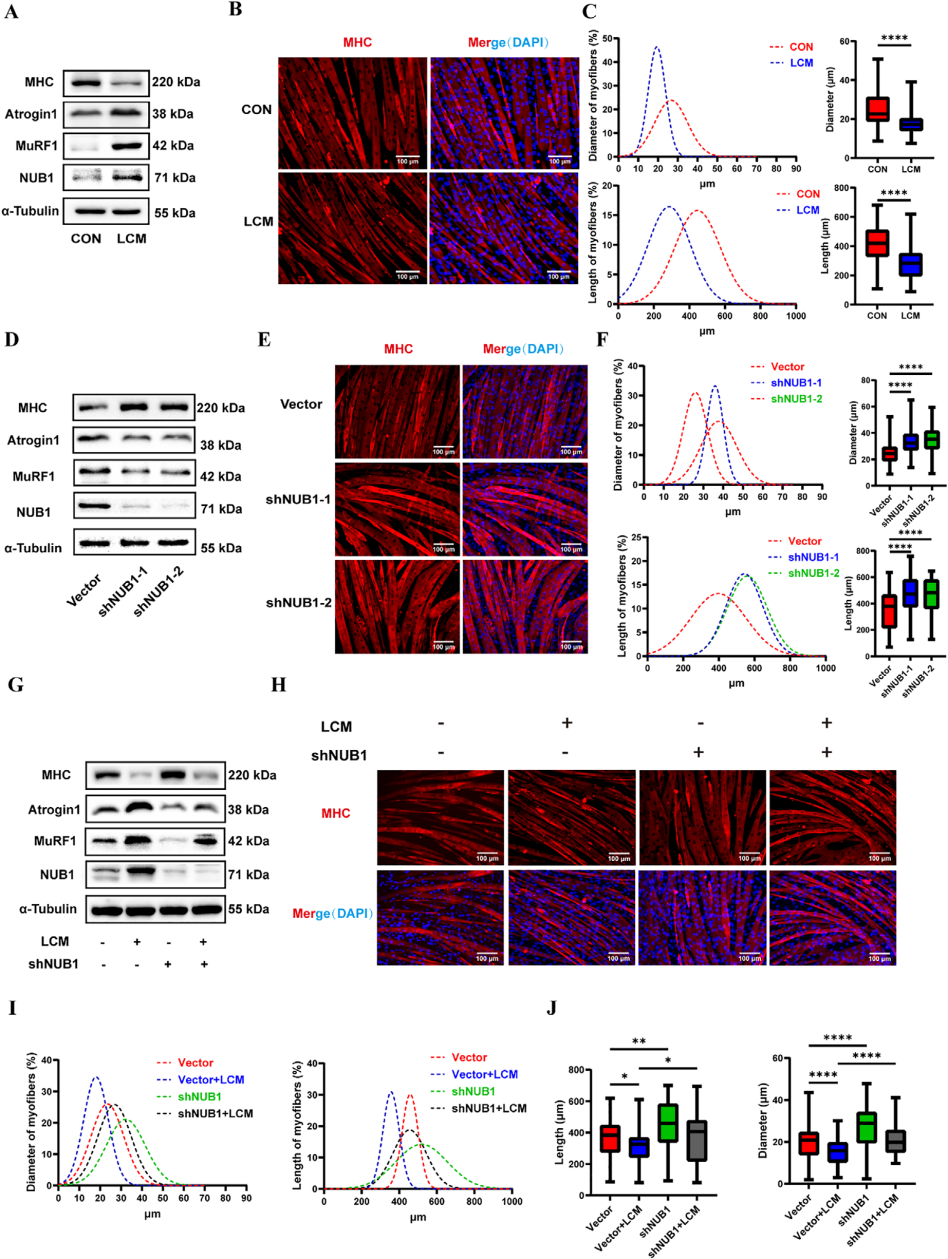
Silencing NUB1 alleviates myotube atrophy in lung cancer cachexia. (A) Western blot analysis of MHC, Atrogin1, MuRF1, and NUB1 in CON and LCM‑treated myotubes (n = 3). (B, C) Immunofluorescence staining of MHC and quantification of myotube diameter and length (scale bar = 100 µm) (n = 3). (D) Western blot analysis of MHC, Atrogin1, MuRF1, and NUB1 in C2C12 cells infected with control (Vector) or NUB1 shRNA (shNUB1‑1, shNUB1‑2) (n = 3). (E, F) Immunofluorescence of MHC and morphometric analysis of myotubes after NUB1 knockdown (scale bar = 100 µm) (n = 3). (G) Western blot analysis of MHC, Atrogin1, MuRF1, and NUB1 in LCM‑treated myotubes with or without NUB1 silencing (n = 3). (H–J) Immunofluorescence detection of MHC and quantification of myotube diameter and length in all groups (scale bar = 100 µm) (n = 3). Data are mean ± SEM from ≥3 independent biological replicates (n = number of independent experiments). Two‐group comparisons used two‐tailed Student's t‐test; multiple groups were compared by one‐way ANOVA with appropriate post‐hoc test. *p < 0.05, **p < 0.01, ***p < 0.001, ****p < 0.0001; ns, not significant.

Rescue experiments further showed that NUB1 knockdown partially blocked LCM‑induced upregulation of Atrogin1 and MuRF1 and reversed the reduction in MHC (Figure 2G; Figure S3C). Accordingly, silencing NUB1 partially restored myotube diameter and length suppressed by LCM (Figure 2H‐J). These results indicated that NUB1 silencing attenuated myotube atrophy *in vitro*.

### 2.3 NUB1 Regulates the NF‐κB Pathway in Lung Cancer Cachexia

To explore the mechanism by which NUB1 regulates muscle atrophy, we reanalyzed the GSE144567 muscle transcriptome dataset from lung cancer cachexia mice [37]. KEGG, GO, and GSEA analyses revealed that NUB1 is closely associated with the NF‑κB signaling pathway (Figure 3A–C). *In vitro*, NUB1 knockdown reduced phospho‑p65 levels and increased IκBα expression, indicating inhibition of the canonical NF‑κB pathway (Figure 3D). NUB1 did not affect non‑canonical NF‑κB signaling (Figure S4A,B), suggesting a specific effect on the classical pathway.

**FIGURE 3:**
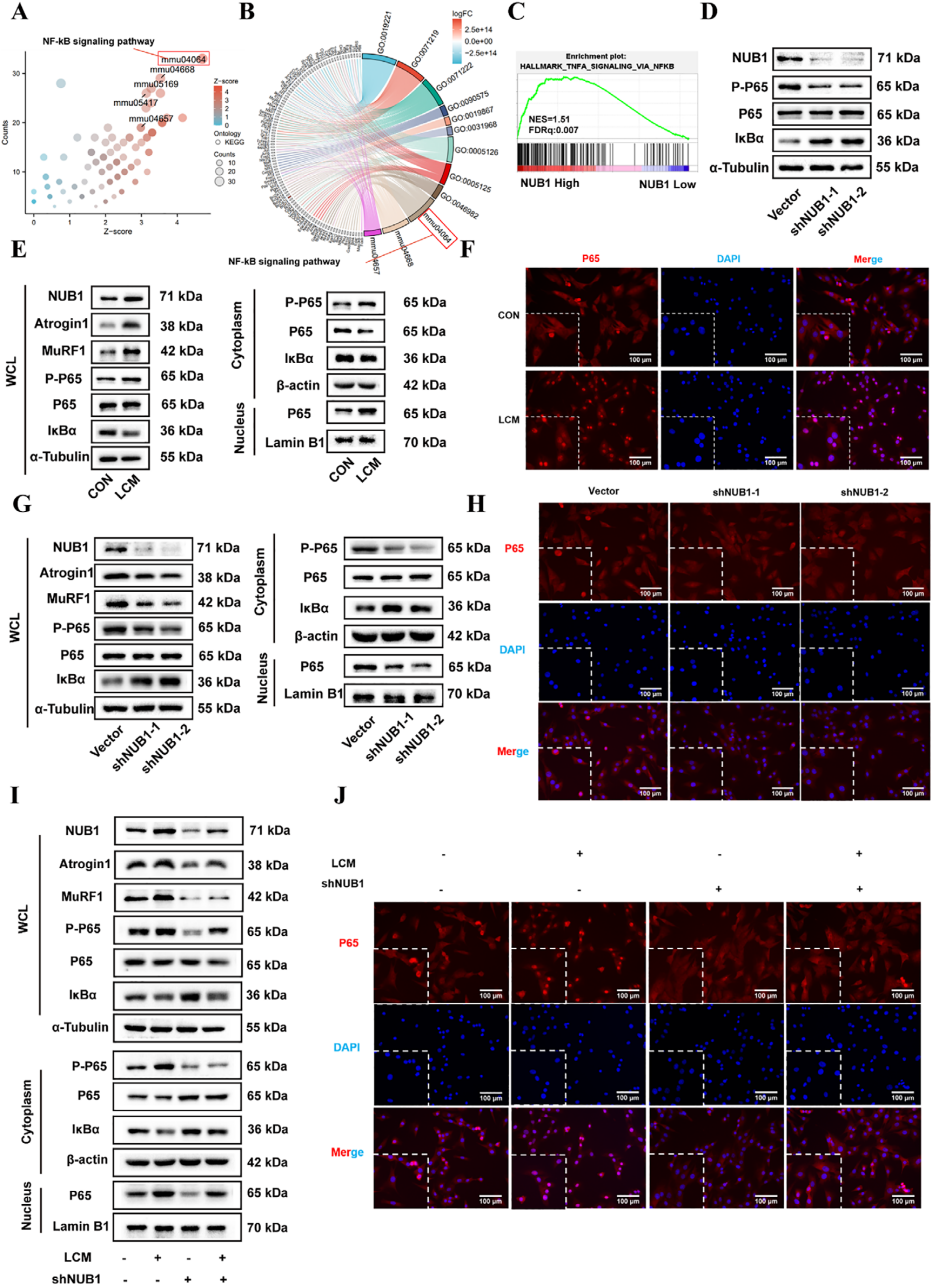
NUB1 regulates the NF‐κB pathway in lung cancer cachexia. (A–C) KEGG, GO, and GSEA analyses of muscle RNA‑seq data from cachexia mice, indicating pathways regulated by NUB1. (D) Western blot analysis of P‐P65, P65, and IκBα after NUB1 silencing (n = 3). (E) Subcellular fractionation and Western blot analysis of NF‑κB components in whole‑cell lysates (WCL), cytoplasm, and nucleus of CON and LCM groups (n = 3). (F) Immunofluorescence staining of P65 nuclear translocation in CON and LCM myotubes (scale bar = 100 µm) (n = 3). (G) Subcellular fractionation and Western blot analysis of NF‑κB proteins in Vector and shNUB1 cells (n = 3). (H) Immunofluorescence showing P65 localization in Vector and shNUB1 cells (scale bar = 100 µm) (n = 3). (I) Subcellular fractionation and Western blot analysis of NF‑κB proteins in Vector, Vector+LCM, shNUB1, and shNUB1+LCM groups (n = 3). (J) Immunofluorescence images of P65 nuclear translocation in the four groups (scale bar = 100 µm) (n = 3). Data are mean ± SEM from ≥3 independent biological replicates (n = number of independent experiments). Two‐group comparisons used two‐tailed Student's t‐test; multiple groups were compared by one‐way ANOVA with appropriate post‐hoc test. *p < 0.05, **p < 0.01, ***p < 0.001, ****p < 0.0001; ns, not significant.

LCM treatment increased phospho‑p65 and decreased IκBα in whole‑cell lysates and cytoplasmic fractions, while promoting p65 nuclear translocation (Figure 3E). Immunofluorescence confirmed enhanced nuclear p65 accumulation in LCM‑treated cells (Figure 3F; Figure S3E). In contrast, NUB1 silencing reduced phospho‑p65, restored IκBα, and decreased nuclear p65 (Figure 3G). Immunofluorescence verified these changes (Figure 3H; Figure S3F). Moreover, NUB1 knockdown attenuated LCM‑induced p65 nuclear translocation (Figure 3I,J; Figure S3G). Together, These data showed that NUB1 silencing inhibited NF‑κB pathway activation in muscle atrophy.

### 2.4 TNFα‐Mediated NF‐κB Activation Rescues NUB1 Effects in Lung Cancer Cachexia *in vitro*

To confirm that NUB1 functions through NF‑κB, we performed rescue experiments using TNFα, a classic NF‑κB activator. In NUB1‑silenced myotubes, TNFα treatment reduced IκBα, restored phospho‑p65, and promoted p65 nuclear translocation (Figure 4A). Immunofluorescence confirmed these effects (Figure 4B; Figure S3H). Functionally, TNFα reversed the NUB1 silencing‑mediated reduction in Atrogin1 and MuRF1 and enhanced MHC degradation (Figure 4C; Figure S3D). MHC staining further showed that TNFα blocked the protective effect of NUB1 knockdown on myotube morphology (Figure 4D–F). These results demonstrated that NUB1 regulated myotube atrophy by controlling NF‑κB signaling.

**FIGURE 4:**
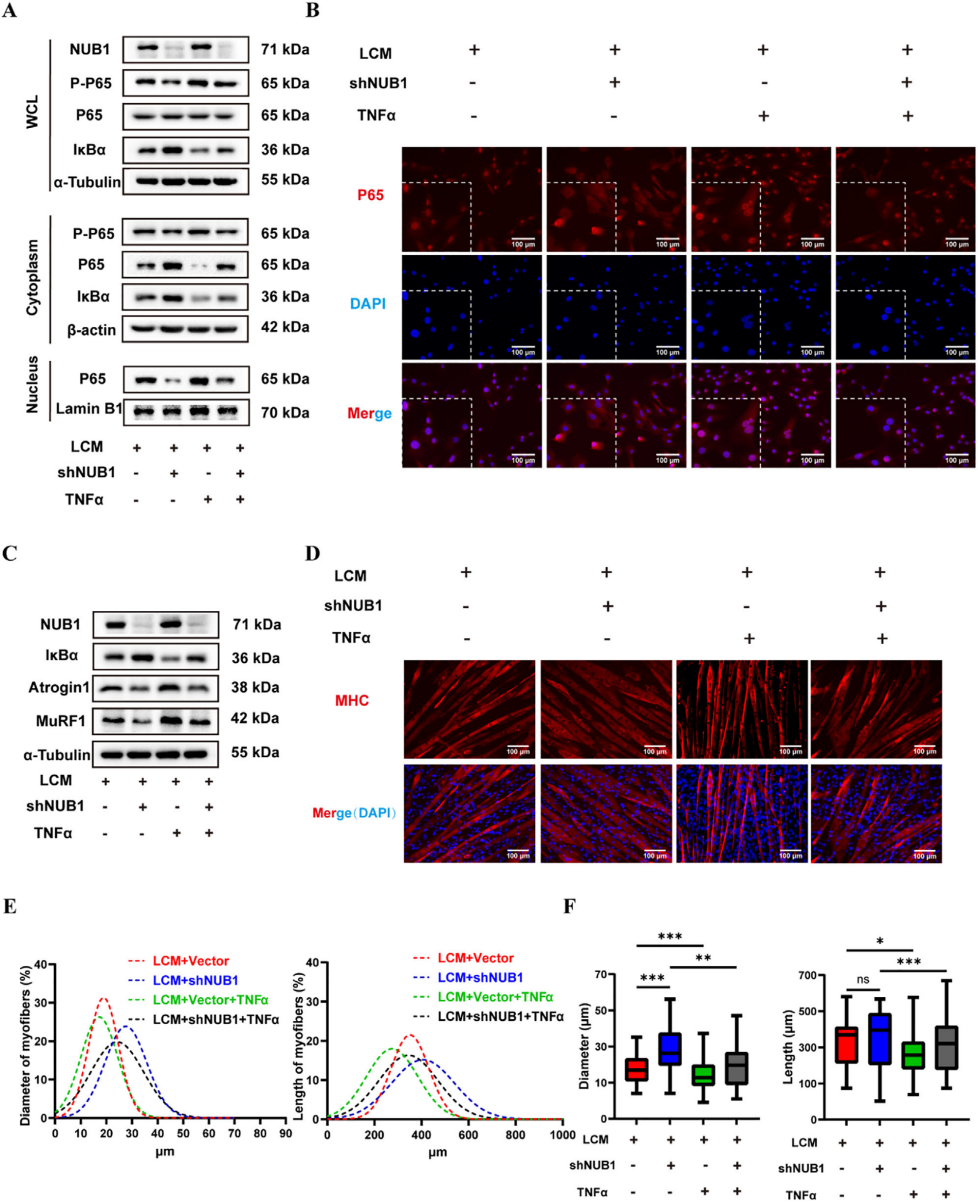
TNFα‐mediated NF‐κB activation rescues NUB1 effects in lung cancer cachexia *in vitro*. (A) Subcellular fractionation and Western blot analysis of NF‑κB pathway proteins in Vector and shNUB1 cells with or without TNFα (25 ng/mL) treatment (n = 3). (B) Immunofluorescence staining of P65 nuclear translocation in Vector, shNUB1, Vector+TNFα, and shNUB1+TNFα groups (scale bar = 100 µm) (n = 3). (C) Western blot analysis of IκBα, MuRF1, Atrogin1, and NUB1 in the four groups (n = 3). (D) Immunofluorescence staining of MHC in all groups (scale bar = 100 µm) (n = 3). (E, F) Quantification of myotube diameter and length from five random fields per group (n = 3). Data are mean ± SEM from ≥3 independent biological replicates (n = number of independent experiments). Two‐group comparisons used two‐tailed Student's t‐test; multiple groups were compared by one‐way ANOVA with appropriate post‐hoc test. *p < 0.05, **p < 0.01, ***p < 0.001, ****p < 0.0001; ns, not significant.

### 2.5 NUB1 Interacts with IκBα and Promotes its Proteasomal Degradation

To investigate how NUB1 affects the NF‐κB pathway and protein degradation, we first examined IκBα, a key inhibitor of NF‐κB, at both mRNA and protein levels following NUB1 silencing. NUB1 knockdown did not alter *Nfkbia* mRNA levels but significantly increased IκBα protein abundance, indicating that NUB1 regulates IκBα post‐translationally (Figure 5A,B). Moreover, neither NUB1 knockdown nor Flag‐NUB1 overexpression notably affected IKKβ or phospho‐IκBα (Ser32/36) protein levels, yet total IκBα levels were substantially changed (Figure S4C–F).

**FIGURE 5:**
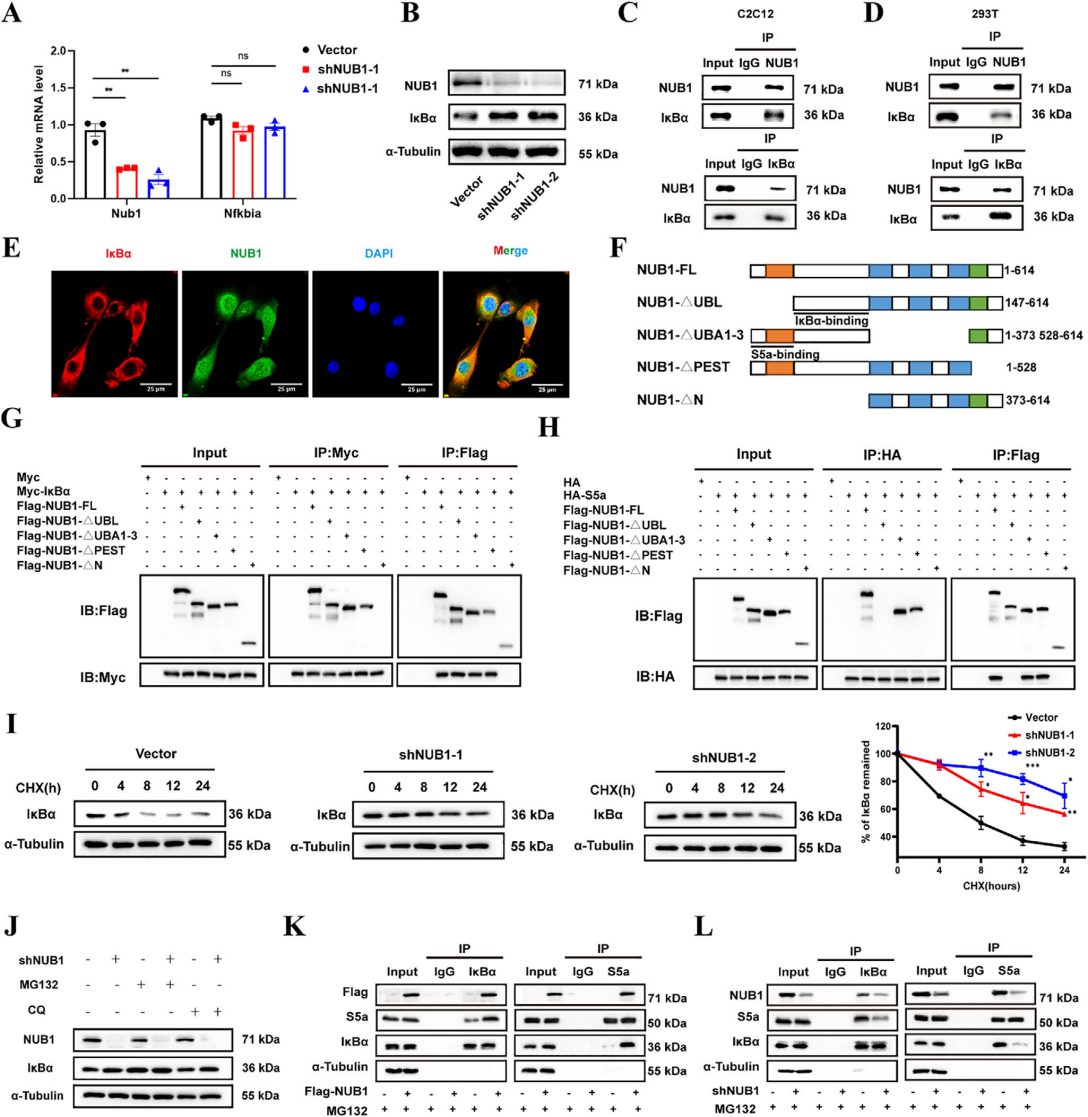
NUB1 interacts with IκBα and promotes its proteasomal degradation. (A) qPCR analysis of *Nfkbia* mRNA in Vector and shNUB1 cells (n = 3). (B) Western blot analysis of IκBα protein after NUB1 silencing (n = 3). (C, D) Endogenous co‑immunoprecipitation (Co‑IP) confirming NUB1‑IκBα interaction in C2C12 and 293T cells (n = 3). (E) Confocal immunofluorescence showing co‑localization of NUB1 and IκBα in C2C12 cells (scale bar = 25 µm) (n = 3). (F) Schematic of full‐length and truncated NUB1 constructs: NUB1‐FL (1–614), NUB1‐△UBL (147–614), NUB1‐△UBA1‐3 (1–373, 528–614), NUB1‐△PEST (1–528), and NUB1‐△N (373–614) (n = 3). (G) Co‑IP assays identifying the IκBα‑binding domain on NUB1 in 293T cells (n = 3). (H) Co‑IP assays characterizing the S5a‑binding domain on NUB1 in 293T cells (n = 3). (I) IκBα protein stability measured by cycloheximide (CHX, 5 µM) chase assay after NUB1 silencing in C2C12 cells (n = 3). (J) Western blot analysis of IκBα in NUB1‑silenced cells treated with MG132 (10 µM) or chloroquine (CQ, 10 µM) in C2C12 cells (n = 3). (K, L) Co‑IP assays showing the effect of NUB1 overexpression or knockdown on IκBα‑S5a interaction in C2C12 cells (n = 3). Data are mean ± SEM from ≥3 independent biological replicates (n = number of independent experiments). Two‐group comparisons used two‐tailed Student's t‐test; multiple groups were compared by one‐way ANOVA with appropriate post‐hoc test. *p < 0.05, **p < 0.01, ***p < 0.001, ****p < 0.0001; ns, not significant.

We next investigated whether NUB1 interacts with IκBα. Co‐immunoprecipitation assays in C2C12 and 293T cells confirmed the interaction between NUB1 and IκBα (Figure 5C,D). Exogenous Co‐IP further showed that NUB1 bound to both wild‐type (Figure S5A) and Ser32/36A mutant IκBα (Figure S5B), and confocal microscopy verified their co‐localization in the cytoplasm (Figure 5E). Functionally, overexpression of NUB1 reduced protein levels of both wild‐type and Ser32/36A IκBα (Figure S5C). TPCA‐1, a specific inhibitor targeting IKK that blocks IκBα phosphorylation at Ser32/36, failed to reverse NUB1‐mediated regulation of IκBα levels in both NUB1‐knockdown and NUB1‐overexpressing C2C12 cells (Figure S5D,E). Together, these results indicated that NUB1 promoted IκBα degradation through a mechanism independent of classical IKK‐mediated Ser32/36 phosphorylation, thereby regulating the IκBα/NF‐κB axis.

We then constructed a series of NUB1 domain‐deleted mutants (Figure 5F). Co‐IP assays showed that the 147–373 amino acid region of NUB1 was required for IκBα binding (Figure 5G). Further studies excluded neddylation, as NUB1 knockdown did not alter IκBα neddylation (Figure S5F), and NEDD8 silencing did not disrupt the NUB1–IκBα interaction (Figure S5G,H). The proteasome subunit S5a bound to the N‐terminal 1–146 region of NUB1, the UBL domain, which was separate from the IκBα‐binding site (Figure 5H).

Cycloheximide chase assays showed that NUB1 depletion stabilized IκBα protein (Figure 5I). Inhibition experiments using MG132 (proteasome inhibitor) and chloroquine (autophagy inhibitor) demonstrated that NUB1 promoted IκBα degradation specifically through the proteasome pathway (Figure 5J). Mechanistically, NUB1 overexpression enhanced the interaction between IκBα and S5a (Figure 5K), whereas NUB1 knockdown reduced this association (Figure 5L). Given that the UBL domain of NUB1 binds S5a and can target IκBα to the proteasome for degradation, we further examined the effect of NUB1 modulation on IκBα ubiquitination. Notably, altering NUB1 expression did not affect IκBα ubiquitination levels (Figure S5I,J), further supporting a unique role of this UBL‐UBA family member in substrate degradation.

In summary, NUB1 acted as an adaptor protein that recruited IκBα to the proteasome for degradation, thereby activating NF‑κB signaling and promoting muscle atrophy.

### 2.6 Silencing NUB1 Ameliorates Muscle Atrophy *in vivo* 

We used adeno‑associated virus (AAV) to silence NUB1 specifically in mouse gastrocnemius muscle. *In vitro* validation confirmed that AAV‐shNUB1 reduced *Nub1* mRNA levels by more than 70% (Figure S6A). *In vivo*, AAV‐mediated knockdown was stably maintained for at least 4 weeks, with fluorescence predominantly restricted to the gastrocnemius muscle (Figure S6B). We further examined *Nub1* mRNA levels in the gastrocnemius (GA) and tibialis anterior (TA) muscles of mice at 1–4 weeks post‐AAV injection (Figure S6C,D). Significant reduction was observed only in the GA muscle at the injection site, confirming that AAV infection remained localized to the site of injection.

Mice were divided into four groups: PBS+AAV‐Vector, LLC+AAV‐Vector, PBS+AAV‐shNUB1, and LLC+AAV‐shNUB1 (Figure 6A). Body weight and final tumor weight were monitored throughout the experiment (Figure 6B; Figure S6E). After 4 weeks, NUB1 silencing partially attenuated tumor‐free body weight loss (Figure 6C) and GA muscle atrophy (Figure 6D), while having no significant effect on TA weight (Figure 6E). Adipose tissue weights (Figure S6F,G) and serum levels of inflammatory cytokines, including IL‐6 (Figure S6H), CXCL1/KC/IL‐8 (Figure S6I), and TNFα (Figure S6J), were also assessed. AAV‐shNUB1 treatment did not significantly change these parameters, suggesting that muscle‐targeted interference had limited systemic effects. Grip strength and endurance tests showed significantly improved muscle function (Figure 6F,G). Histological analysis revealed that NUB1 knockdown increased myofiber cross‐sectional area (Figure 6H–J). At the protein level, NUB1 silencing suppressed Atrogin1, MuRF1, and NF‐κB activation, while restoring MHC expression (Figure 6K). These results indicated that muscle‐specific NUB1 silencing mitigated muscle mass loss, weakness, and atrophy in lung cancer cachexia.

**FIGURE 6:**
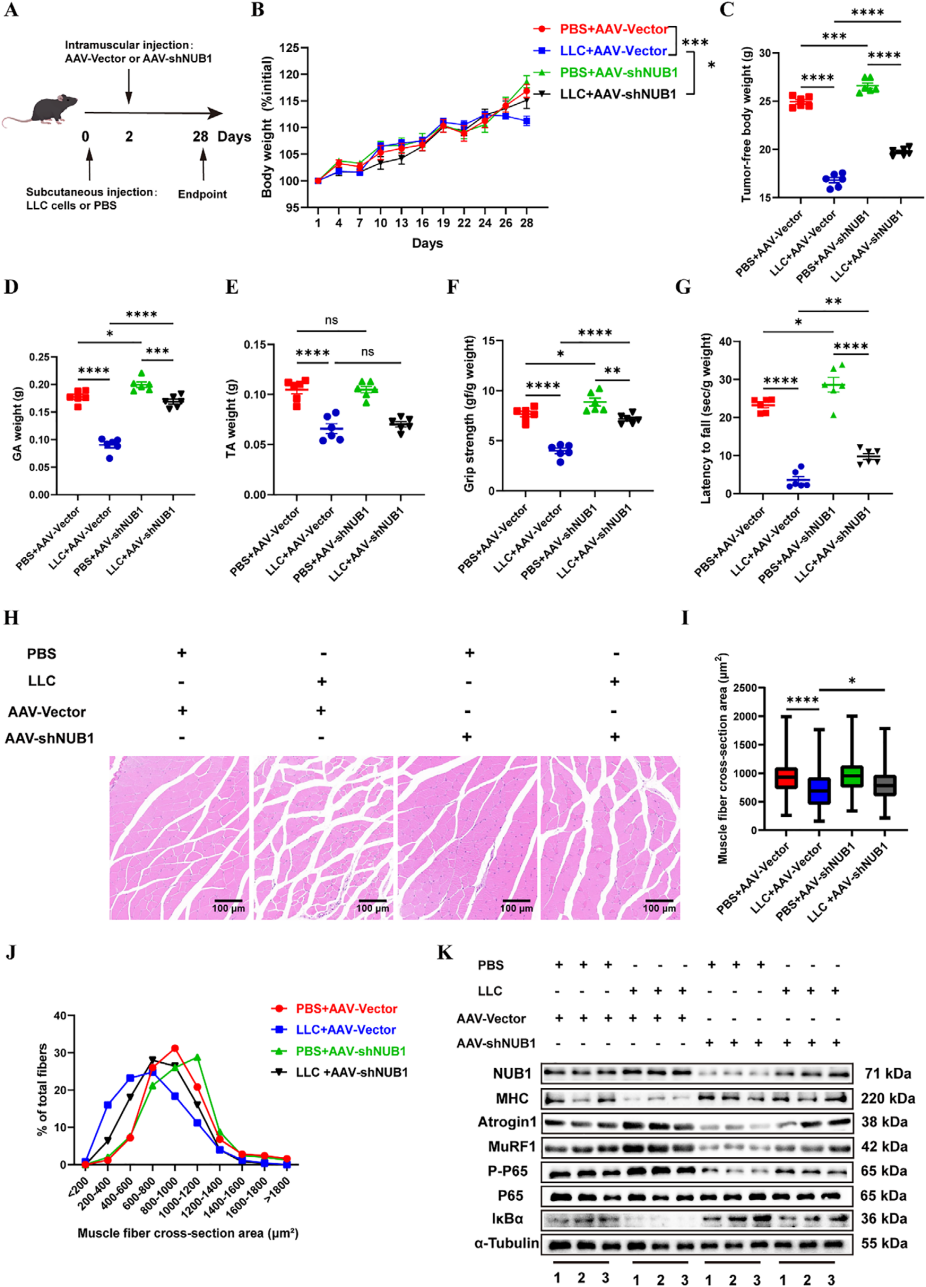
Silencing NUB1 ameliorates muscle atrophy of lung cancer cachexia *in vivo*. (A) Schematic of AAV‑shNUB1 injection into gastrocnemius muscles of LLC tumor‑bearing mice. (B) Body weight curves of PBS+AAV‑Vector, LLC+AAV‑Vector, PBS+AAV‑shNUB1, and LLC+AAV‑shNUB1 mice (n = 6). (C, D) Tumor‑free body weight and gastrocnemius muscle weight in each group (n = 6). (E) Tibialis anterior weight in four groups (n = 6). (F) Forelimb grip strength normalized to body weight (n = 6). (G) Rotarod performance expressed as latency to fall normalized to body weight (n = 6). (H) Representative H&E staining of gastrocnemius muscle (scale bar = 100 µm). (I, J) Quantification of myofiber cross‑sectional area (50 fibers per field, 5 fields per group). (K) Western blot analysis of NUB1, MHC, Atrogin1, MuRF1, P‑P65, P65, and IκBα in gastrocnemius tissues (n = 3). Data are mean ± SEM from ≥3 independent biological replicates (n = number of independent experiments). Two‐group comparisons used two‐tailed Student's t‐test; multiple groups were compared by one‐way ANOVA with appropriate post‐hoc test. *p < 0.05, **p < 0.01, ***p < 0.001, ****p < 0.0001; ns, not significant.

### 2.7 Targeting NUB1 Alleviates Muscle Atrophy via Modulating the NF‐κB Pathway *in vivo*

To verify the NUB1‑NF‑κB axis *in vivo*, we performed rescue experiments using TNFα. Mice were divided into five groups: PBS control, LLC+AAV‑Vector+PBS, LLC+AAV‑shNUB1+PBS, LLC+AAV‑Vector+TNFα, and LLC+AAV‑shNUB1+TNFα (Figure 7A).

**FIGURE 7:**
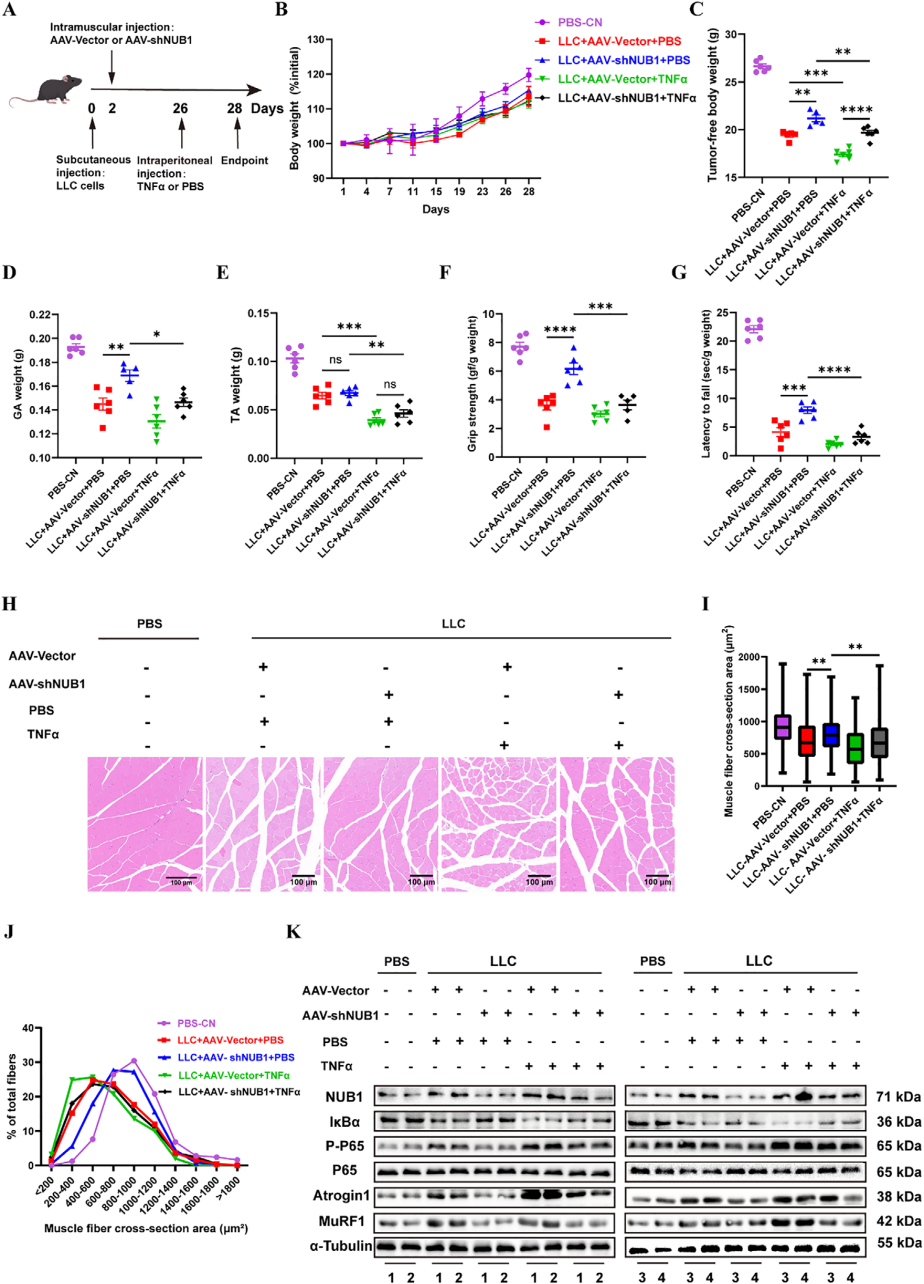
Targeting NUB1 alleviates muscle atrophy via modulating the NF‐κB pathway *in vivo*. (A) Schematic of TNFα administration in LLC tumor‑bearing mice after AAV‑shNUB1 treatment. (B) Body weight curves of five experimental groups (n = 6). (C, D) Tumor‑free body weight and gastrocnemius weight in each group (n = 6). (E) Tibialis anterior weight in five groups (n = 6). (F) Normalized forelimb grip strength (n = 6). (G) Normalized rotarod endurance (n = 6). (H) Representative H&E staining of gastrocnemius muscle. (I, J) Quantification of myofiber cross‑sectional area (50 fibers/field, 250 fibers total). (K) Western blot analysis of NUB1, IκBα, P‑P65, P65, MuRF1, and Atrogin1 in gastrocnemius tissues (n = 4). Data are mean ± SEM from ≥3 independent biological replicates (n = number of independent experiments). Two‐group comparisons used two‐tailed Student's t‐test; multiple groups were compared by one‐way ANOVA with appropriate post‐hoc test. *p < 0.05, **p < 0.01, ***p < 0.001, ****p < 0.0001; ns, not significant.

TNFα treatment partially reversed the improvements in body weight (Figure 7B,C),and gastrocnemius muscle mass (Figure 7D) induced by NUB1 silencing. Consistently, AAV‐shNUB1 had minimal effects on tumor weight (Figure S6K) and TA muscle weight (Figure 7E). Similarly, TNFα blocked the beneficial effects of NUB1 knockdown on grip strength and endurance (Figure 7F,G) and reduced myofiber cross‐sectional area (Figure 7H–J). In muscle tissue, TNFα abolished the NUB1 silencing‐mediated increase in IκBα and reactivated NF‐κB signaling (Figure 7K). These results confirmed that NUB1 promotes muscle atrophy by activating the NF‐κB pathway *in vivo*.

## 3 Discussion

Cancer cachexia represents a progressive metabolic syndrome characterized by irreversible skeletal muscle wasting, which profoundly compromises physical performance, treatment tolerance, and long‐term prognosis in patients with advanced malignancies [43]. Although current pharmacological interventions, such as anamorelin and megestrol acetate, offer modest symptomatic relief by stimulating appetite, they fail to fundamentally reverse muscle catabolism [44, 45, 46]. Therefore, identifying the molecular drivers of muscle loss is a critical step for developing effective therapies. In this study, we screened and validated NUB1 as a previously unveiled upstream regulator that promotes skeletal muscle atrophy in lung cancer cachexia, providing a potential therapeutic target for mitigating cancer‐associated muscle depletion.

The ubiquitin‐proteasome system (UPS) is well established as a central mechanism of cancer‐associated muscle proteolysis and serves as the principal molecular system responsible for the degradation of myofibrillar proteins [47, 48]. Specifically, the muscle‐specific E3 ubiquitin ligases, Atrogin1 and MuRF1, serve as core downstream effectors that directly mediate myofibrillar destruction and muscle dysfunction [49]. Multiple pathways and regulatory factors, such as the NF‐κB, FOXO, and p38 MAPK‐C/EBPβ signaling, have been reported to modulate UPS activity in cancer‐induced muscle wasting [39, 50, 51, 52]. Here, we demonstrated that NUB1 was significantly upregulated in skeletal muscle from Lewis lung carcinoma (LLC) cachexia models. Notably, silencing NUB1 both *in vitro* and *in vivo* attenuated NF‐κB signaling, thereby suppressing Atrogin1 and MuRF1 expression, indicating that NUB1 functions as a key upstream activator of UPS‐mediated muscle wasting.

NUB1 belongs to the ubiquitin‐like‐ubiquitin‐associated (UBL‐UBA) protein family, which modulates protein modification and proteostasis. Traditionally, NUB1 facilitates substrate degradation by cooperating with ubiquitin‐like proteins, including NEDD8 or FAT10, or by directly targeting substrates to the proteasome for degradation [25, 53]. For instance, it promotes the ubiquitin‐independent degradation of FAT10‐conjugated substrates by docking with 26S proteasome subunits Rpn1 and Rpn10 (S5a) [24], or modulates tau proteostasis by facilitating either proteasomal degradation or autophagy‐lysosomal clearance [54]. In other pathological contexts, NUB1 acts as a tumor suppressor in hepatocellular carcinoma by promoting PCNA degradation via the neddylation pathway [26], or inhibits NF‐κB signaling in rheumatoid arthritis by attenuating CUL1 neddylation [55]. However, in lung cancer cachexia, we found that NUB1 regulates NF‐κB signaling and muscle atrophy by modulating IκBα protein homeostasis.

Mechanistically, NUB1 bound to both the proteasome subunit S5a and IκBα, forming a complex that brought IκBα into close proximity with the 26S proteasome. Overexpression of NUB1 significantly strengthened the interaction between IκBα and S5a, whereas depletion of NUB1 markedly weakened it, suggesting that NUB1 acted as an essential adaptor to deliver IκBα to the proteasome. This mechanism mirrored the established role of NUB1 in promoting TRF1 degradation [23], where it similarly functioned as a bridging molecule that connected substrates to S5a for proteasomal breakdown. However, in contrast to the NEDD8‐dependent pathway involved in TRF1 degradation, this IκBα degradation process occurred independently of NEDD8 modification, defining a distinct, tissue‐specific mechanism by which NUB1 drove muscle wasting in lung cancer cachexia.

Aberrant NF‑κB activation, driven by pro‑inflammatory cytokines such as TNFα or IL‑6, is a known driver of muscle loss in cancer cachexia [56, 57]. However, how NF‐κB signaling remains persistently active within muscle cells or tissue warrants further investigation. We demonstrated that NUB1 links NF‐κB signaling to the downstream muscle‐specific E3 ligases (Atrogin1/MuRF1), thereby extending the classic cytokine‐driven view of NF‐κB activation by uncovering an intracellular protein degradation pathway. Collectively, NUB1 serves to refine and complement existing mechanisms underlying NF‐κB‐driven muscle wasting, highlighting NUB1 as a critical mediator of muscle catabolism and underscoring the mechanistic basis for developing targeted therapies against cancer cachexia.

The translational potential of targeting NUB1 was strongly supported by the significant mitigation of cachexia symptoms via muscle‐specific AAV silencing. This finding was consistent with recent advances in engineered muscle‐tropic AAV capsids. MyoAAV, a capsid variant identified through directed evolution using the DELIVER platform, displays enhanced binding affinity to integrin heterodimers and muscle‐specific receptors, enabling highly efficient transduction of skeletal and cardiac muscle with minimal off‐target effects [58, 59] Therefore, refining AAV capsids to better target muscle tissue could further enhance the therapeutic benefit. Furthermore, emerging imaging techniques such as Fourier ptychographic microscopy may enable better assessment of structural complexity changes in muscle tissue [60].Moreover, the interactions of NUB1 with IκBα or the proteasome subunit S5a presented specific targets for therapeutic intervention, supporting the development of small‐molecule inhibitors through structure‐based screening or targeted delivery systems, such as MyomP1‐functionalized lipid nanoparticles, aimed at disrupting these complexes [61]. Importantly, this muscle‐focused approach may circumvent the systemic toxicities associated with broad NF‐κB inhibition. Conventional NF‐κB inhibitors, including bortezomib and IKKβ inhibitors such as ML120B and BMS‐345542, face dose‐limiting toxicities including peripheral neuropathy, hepatotoxicity, and compromised immune surveillance [62]. Systemic NF‐κB blockade weakens immune function and may impair antitumor immunity. Thus, tissue‐specific strategies that selectively target pathological NF‐κB activation in skeletal muscle are of critical importance [63]. Finally, while our study uncovered the NUB1‐IκBα‐NF‐κB regulatory mechanism in lung cancer cachexia, the central role of NF‐κB hyperactivation in driving muscle proteolysis was well documented across diverse malignancies [14]. Thus, the molecular axis established here provides a conceptual foundation that may offer NUB1‐targeted strategies in other cancer cachexia environments.

In conclusion, this study elucidated a formerly unknown mechanism driving muscle wasting in lung cancer cachexia. Upregulation of NUB1 in skeletal muscle represented a critical mediator of tumor‐muscle crosstalk that promoted progressive atrophy and functional decline via sustained NF‐κB pathway activation. By facilitating IκBα proteasomal degradation through interaction with the proteasome subunit S5a, NUB1 enhanced NF‐κB‐mediated transcription of *Atrogin1* and *MuRF1*, accelerating muscle protein breakdown. Targeting the NUB1/IκBα/NF‐κB axis therefore represented a promising therapeutic strategy to counteract lung cancer–induced muscle loss (Figure 8). These findings deepen our mechanistic understanding of cachexia pathogenesis and lay the groundwork for developing NUB1‐targeted interventions to preserve skeletal muscle mass in patients with malignancies.

**FIGURE 8:**
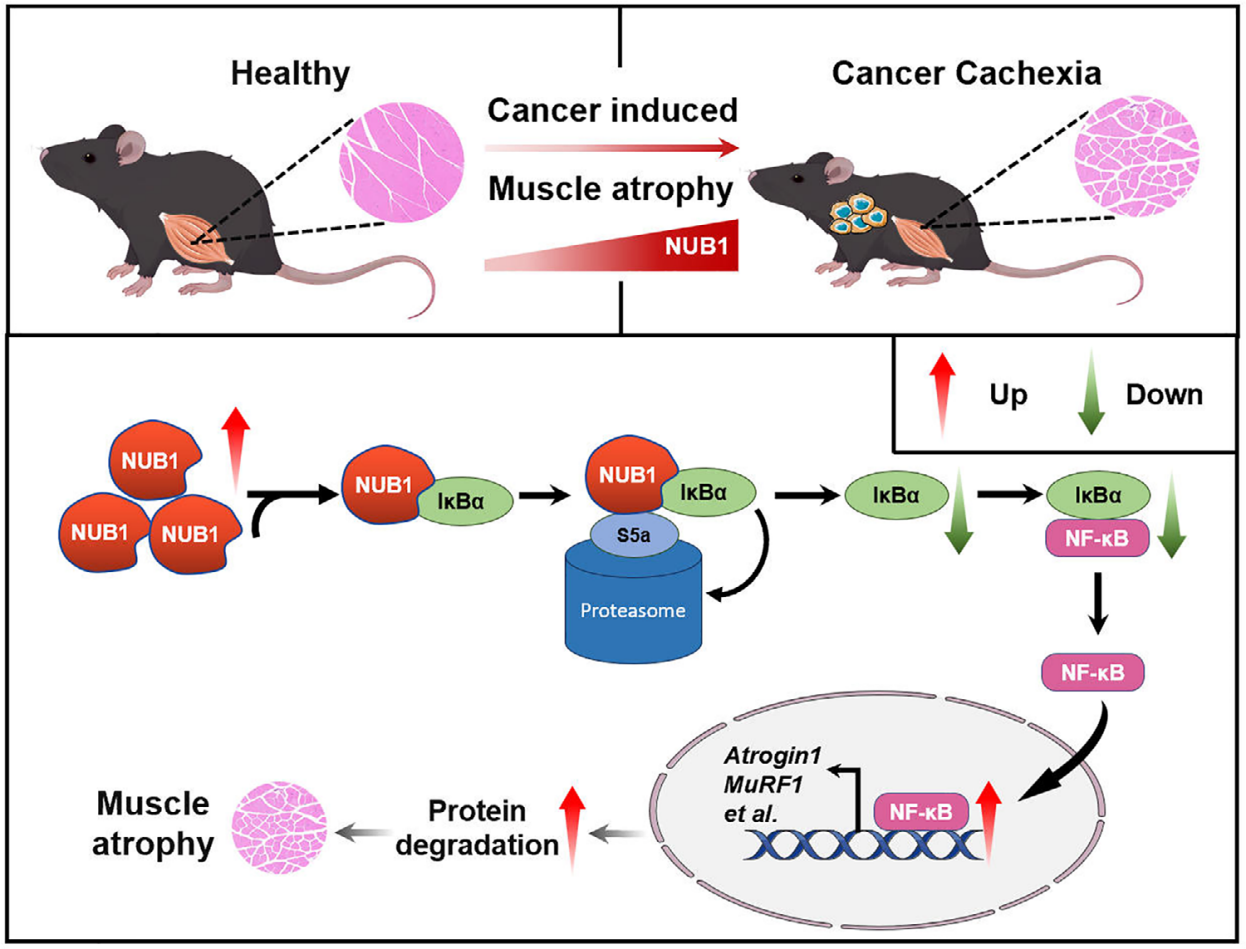
NUB1‐mediated regulation of skeletal muscle atrophy in lung cancer cachexia. This diagram illustrated the molecular mechanism by which NUB1 regulated skeletal muscle atrophy in lung cancer cachexia. NUB1 was significantly upregulated under cachectic conditions, where it bound to IκBα and recruited the proteasomal subunit S5a. This interaction promoted proteasomal degradation of IκBα, thereby enabling NF‐κB translocation into the nucleus. Nuclear NF‐κB subsequently enhanced the transcription of E3 ubiquitin ligases Atrogin1 and MuRF1, which accelerated muscle protein degradation and ultimately led to skeletal muscle atrophy. These findings highlighted the critical function of NUB1 in muscle mass maintenance and identified NUB1 as a promising therapeutic target for cancer cachexia.

## 4 Experimental Section

### 4.1 Animal assays

C57BL/6J male mice were purchased from Hubei Beinte Biotechnology Co., Ltd. (Wuhan, China). All animal experiments and protocols were conducted under the ethical guidelines of Huazhong University of Science and Technology (IACUC Number:4642). The mice were housed at a standard temperature and in a light‐controlled environment.

### 4.2 Cell culture and drug treatments

C2C12 cells (SCSP‐505, the Cell Bank of the Chinese Academy of Sciences, Shanghai, China), LLC cells (Lewis Lung carcinoma, ATCCCRL‐ 1642, ATCC, USA) and HEK293T cells (ATCCCRL‐3216, ATCC, USA) were cultured in Dulbecco's modified Eagle's medium (DMEM) with 10% fetal bovine serum (FBS) and 1% penicillin and streptomycin at 37°C supplemented with 5% CO2. For C2C12 differentiation, when cell confluence reached 70%, the medium was switched into differentiation medium (DMEM containing 2% horse serum) for 4 days. Cells were treated with cycloheximide (CHX, 5 µM) for 0, 4, 8, 12, or 24 h. For inhibition of the proteasome and autophagy–lysosome pathways, cells were exposed to 10 µM MG132 or CQ for 10–12 h [64, 65]. Recombinant mouse TNFα was reconstituted in sterile water with 5% trehalose in PBS to a stock concentration of 200 µg/mL and applied at 25 ng/mL for 24 h [66, 67]. Additionally, cells were incubated with 5 µM TPCA‐1 for 24 h [68]. The catalog numbers of the reagents are listed in Table S1.

### 4.3 Cancer cachexia models *in vitro*

LLC cells were seeded in 10 cm dishes and grown to >90% confluence in complete medium. After two PBS washes, cells were serum‐starved in FBS‐free DMEM for 48 h. The supernatant was collected, centrifuged at 500 × g for 5 min, and mixed with fresh complete medium at a volume ratio of 1:3 to generate LLC‐conditioned medium (LCM). An equal volume of complete medium was used as the control medium (CON). Both media were used immediately. Following differentiation to mature myotubes, C2C12 cells were incubated for 48 h with freshly prepared LCM. Phase‐contrast microscopy revealed pronounced myotube thinning and increased cell death; concomitant reductions in myosin heavy chain and induction of atrophy markers (Atrogin1, MuRF1) confirmed successful establishment of the lung‐cancer cachexia muscle‐atrophy model for subsequent analyses.

### 4.4 Cell infection and transfection

Lentiviral infection: Early‐passage C2C12 myoblasts seeded at 45–55% confluence were infected with NUB1‐shRNA lentivirus (Genechem) in serum‐free DMEM plus infection enhancer for 8–10 h. After medium replacement with complete DMEM, cells were cultured for 48–72 h and monitored by GFP fluorescence. Puromycin was added when cells reached ∼80% confluence; cultures with ≥75% GFP‐positive cells were validated for silencing efficiency and frozen as stable stocks for subsequent experiments. Plasmid transfection: The following plasmids were constructed: Flag‐NUB1‐FL, Flag‐NUB1‐△UBL, Flag‐NUB1‐△UBA1‐3, Flag‐NUB1‐△PEST, Flag‐NUB1‐△N, Myc‐IκBα, Myc‐IκBα (Ser32A/Ser36A), and HA‐S5a (GENEWIZ). Cells were cultured to 60% confluence, followed by medium replacement with fresh complete medium. Transfection complexes were prepared using Lipo8000 transfection reagent (Beyotime) and added to cells. Cells were harvested 48 h post‐transfection for subsequent experiments. siRNA transfection: C2C12 cells were seeded in six‐well plates and grown to ∼60% confluence in complete medium. After two PBS washes, 1 mL fresh complete medium was added per well. The transfection mixture—125 µL Opti‐MEM containing 5 µL of 20 µM siRNA and 4 µL Lipo8000 transfection reagent (Beyotime)—was prepared, mixed gently, and overlaid dropwise. After 48 h, cells were harvested to assess target gene silencing efficiency. The effective target sequences employed are shown in Table S2.

### 4.5 Real time PCR

RNA isolation was performed by homogenization of muscle samples or C2C12 cells in MagZol reagent (Magen) followed by extraction with chloroform and precipitation with isopropanol. 1 µg of total RNA was reverse transcribed into cDNA using the HiScript II Q RT SuperMix for qPCR (Vazyme) according to the manufacturer guidelines. Quantitative real time PCR was performed with the qTOWER^3^ system (Analytik Jena) using the ChamQ Blue Universal SYBR qPCR Master Mix (Vazyme). The mRNA levels were calculated by the 2^−ΔΔCT^ method with the level of GAPDH as the internal control. The primers used for real time PCR are listed in Table S3.

### 4.6 Western blotting

Snap frozen muscles or C2C12 cells were homogenized in ice‐cold RIPA buffer (Beyotime) supplemented with protease inhibitors (Selleck), phosphatase inhibitors (Selleck) and PMSF, and centrifuged for 5 min at 12 000g at 4°C. Protein concentration was measured via a BCA assay (ThermoFisher). Samples were mixed in a 4:1 ratio with 5× SDS‐PAGE Loading Buffer (Servicebio), denatured for 10 min at 100°C, and 30 µg of protein was loaded to an 8–12.5% SDS‐PAGE gel. Thereafter, proteins were transferred to a PVDF Western Blotting Membranes (Roche) using the Transblot turbo transfer system (Biorad). Membranes were blocked with 5% skimmed milk for 1.5h at room temperature and then incubated with primary and secondary antibodies. The densitometry of the western blots was detected by The ChemiDoc XRS+ Gel Imaging System (Biorad). The primary antibodies used are listed in Table S4.

### 4.7 Immunofluorescence staining

C2C12 cells were seeded, rinsed three times with PBS, fixed with 4% paraformaldehyde for 40 min, and permeabilized/blocked with 5% BSA + 0.5% Triton X‐100 for 1 h. Primary antibodies were applied at 4°C overnight, followed by three PBS washes. Fluorophore‐conjugated secondary antibodies were added for 1 h at 37°C in the dark, washed three times, and nuclei were stained with DAPI‐mounted medium for 15 min before imaging.

### 4.8 Co‐Immunoprecipitation

Cells were scraped into 1 mL ice‐cold PBS, pelleted, washed twice with PBS, and lysed in 550 µL lysis buffer on ice for 30 min. Magnetic beads (20 µL) were washed twice with 0.5% PBST and once with RIPA buffer. After centrifugation (12 500 g, 4°C, 6 min), 500 µL lysate was added to the washed beads and incubated at room temperature for 1–2 h. The lysate was then split into two tubes (200 µL with IgG, 300 µL with target antibody) and incubated at 4°C overnight. Beads were washed thrice with RIPA buffer, resuspended in 100 µL lysis buffer plus 25 µL 5× loading buffer, boiled for 10 min, and stored at –80°C for SDS‐PAGE.

### 4.9 Adeno‐associated virus (AAV) intramuscular injection

AAV were purchased from Shandong Vigene Biosciences Co., Ltd. (Jinan, China). AAV‐shNUB1 and AAV‐Vector were diluted in sterile PBS to the desired volume. Mice were shaved on both hind limbs, and 50 µL of virus solution (2×10^1^ ^1^ vg) was injected into each gastrocnemius muscle. After 1–4 weeks, mice were shaved again, anesthetized with 150 µL of 10 µg/mL pentobarbital, and injected intraperitoneally with D‐Luciferin substrate. After 10 min, bioluminescence was imaged.

### 4.10 Cancer cachexia model *in vivo*

C57BL/6J male mice (7‐8 weeks) were acclimated for one week and then randomly assigned to ten groups. 5×10^6^ LLC cells were suspended in 100 µL PBS and injected subcutaneously into the right axilla region of each mouse. Tumor‐bearing mice were monitored every other day for body weight, tumor size, and activity. Endpoints were defined per widely accepted murine cachexia criteria: ≥10% body weight loss (tumor‐free), muscle wasting, and functional decline [69, 70]. Tumor burden was monitored to ensure humane limits were not exceeded.

On day 28, mice were anesthetized with pentobarbital and sacrificed. Tissues including tibialis anterior, gastrocnemius, gonadal fat, and inguinal fat were collected. Some tissues were fixed in 4% paraformaldehyde, while others were snap‐frozen in liquid nitrogen and stored at –80°C. Group assignments were as follows: (1) PBS‐CN: 100 µL PBS injected subcutaneously; (2) LLC‐TB: 100 µL LLC cells injected subcutaneously; (3) PBS+AAV‐Vector: subcutaneous PBS followed by bilateral gastrocnemius injection of 50 µL AAV‐Vector 2 days later; (4) LLC+AAV‐Vector: subcutaneous LLC cells followed by bilateral gastrocnemius injection of 50 µL AAV‐Vector 2 days later; (5) PBS+AAV‐shNUB1: subcutaneous PBS followed by bilateral gastrocnemius injection of 50 µL AAV‐shNUB1 2 days later; (6) LLC+AAV‐shNUB1: subcutaneous LLC cells followed by bilateral gastrocnemius injection of 50 µL AAV‐shNUB1 2 days later; (7) LLC+AAV‐Vector+PBS: group 4 with intraperitoneal PBS 48 h before sacrifice; (8) LLC+AAV‐shNUB1+PBS: group 6 with intraperitoneal PBS 48 h before sacrifice; (9) LLC+AAV‐Vector+TNFα: group 4 with intraperitoneal recombinant TNFα (100 ng/g) 48 h before sacrifice; (10) LLC+AAV‐shNUB1+TNFα: group 6 with intraperitoneal recombinant TNFα (100 ng/g) 48 h before sacrifice.

### 4.11 Grip Strength and Endurance Tests

Forelimb grip strength was measured using a YLS‐13A (Yiran Biotechnology, China) digital grip strength meter. The device was leveled and zero‐calibrated. Each mouse was gently transferred to the platform, grasped by the tail, and pulled back at a constant speed until it released its grip. The maximum force displayed on the screen was recorded. Total forelimb and hindlimb grip strength was measured separately, and hindlimb strength was calculated by subtraction. Each mouse was tested 10 times, and the mean value was used for statistical analysis [71]. Relative grip strength was calculated as absolute force (gf) divided by body mass (g) and expressed as gram‐force per gram (gf/g). Muscle endurance and motor ability were assessed using a YLS‐4C (Zhenghua, China) rotarod fatigue tester. Mice were first trained to acclimate to the rotating rod. The device was set at 35 rpm with a maximum trial duration of 15 min. Each mouse was placed on the accelerating rod, and latency to fall was recorded and used for statistical analysis [72]. Each group was normalized to body mass (latency / mass) to yield relative endurance in seconds per gram(sec/g).

### 4.12 Histological Analysis

Tissues including gastrocnemius and tibialis anterior were fixed in 4% paraformaldehyde, embedded, sectioned, and stained with hematoxylin and eosin (H&E). Histomorphology was analyzed by light microscopy. For muscle analysis, five random fields per group were imaged; 50 fibers per field (250 total) were measured in ImageJ to obtain cross‐sectional area (CSA) for statistical analysis.

### 4.13 Serum Inflammatory Cytokine Assessment

Serum was collected from AAV‐treated LLC tumor‐bearing mice. Serum concentrations of IL‐6, CXCL1/KC (IL‐8), and TNFα were quantified by ELISA using the High Sensitivity Mouse IL‐6 ELISA Kit (Elabscience), Mouse CXCL1/KC (IL‐8) ELISA Kit (Solarbio), and Mouse TNFα ELISA Kit (Biorbyt), respectively.

### 4.14 RNA Sequencing and Data Analysis

Publicly available mouse skeletal‐muscle RNA‐seq datasets for pancreatic KIC (GSE226898) [36], colon C26 (GSE251864) [38], Lewis lung carcinoma LLC (GSE144567) [37], and NSCLC (GSE107470) [34, 35] cachexia models were downloaded from GEO. Differentially expressed genes (DEGs) were identified with DESeq2 (*p* < 0.005, |log2FC| ≥ 1.5) and intersected with the Reactome “metabolism of proteins” gene set (https://reactome.org/PathwayBrowser/#/R‐MMU‐392499&DTAB = MT), yielding four genes including NUB1. For LLC cachexia datasets, KEGG and GO enrichment analyses were performed using clusterProfiler (*p* < 0.05, q < 0.05). GSEA was also conducted, with significant gene sets defined as FDR < 0.25 and |NES| ≥ 1.5.

### 4.15 Statistical Analysis

All experiments were performed independently at least three times to ensure reproducibility. Data are presented as mean ± SEM. Comparisons between two groups were evaluated using two‐tailed Student's t‐test; multiple groups were compared by one‐way ANOVA followed by the appropriate post‐hoc test. All statistical analyses and graphs were generated with GraphPad Prism 10. Statistical significance was set at *p* < 0.05 and is indicated as *p < 0.05, **p < 0.01, ***p < 0.005, ****p < 0.001; ns denotes not significant (*p* ≥ 0.05).

## Author Contributions

Y.H., Y.X., and H.Y. initiated the study and designed the experiments. Y.H. performed most of the experiments, conducted statistical analysis, prepared figures, and drafted the manuscript. W.L. provided scientific feedback and guidance throughout the study. J.W., T.Y., D.T., D.M., W.L., X.D., J.L., and Y.J. assisted with animal experiments and *in vitro* experiments. G.H. provided valuable suggestions. X.Z. and H.Y. supervised the study, provided overall guidance, and ensred the scientific rigor of the work. All authors have thoroughly reviewed, critically revised, and endorsed the final version of the manuscript.

## Funding

National Natural Science Foundation of China (No. 81972630 and 82372842), the Shenzhen Medical Research Fund (No. B2302054), and the Sanming Project of Medicine in Shenzhen (No. SASM202201024).

## Conflicts of Interest

The authors declare no conflicts of interest.

## Data Availability

Bioinformatic data used in this study were obtained from the Gene Expression Omnibus database and the Reactome pathway database. Additional data generated during the study are available from the corresponding author upon reasonable request.

## References

[1] J. M. Argilés , F. J. López‐Soriano , B. Stemmler , and S. Busquets , “Cancer‐associated Cachexia — Understanding the Tumour Macroenvironment and Microenvironment to Improve Management,” Nature Reviews Clinical Oncology 20, no. 4 (2023): 250–264, 10.1038/s41571-023-00734-5. 36806788

[2] F. Bray , M. Laversanne , H. Sung , et al., “Global Cancer Statistics 2022: GLOBOCAN Estimates of Incidence and Mortality Worldwide for 36 Cancers in 185 Countries,” CA: A Cancer Journal for Clinicians 74, no. 3 (2024): 229–263, 10.3322/caac.21834. 38572751

[3] M. Berriel Diaz , M. Rohm , and S. Herzig , “Cancer Cachexia: Multilevel Metabolic Dysfunction,” Nature Metabolism 6, no. 12 (2024): 2222–2245, 10.1038/s42255-024-01167-9. 39578650

[4] H. Nishikawa , M. Goto , S. Fukunishi , A. Asai , S. Nishiguchi , and K. Higuchi , “Cancer Cachexia: Its Mechanism and Clinical Significance,” International Journal of Molecular Sciences 22, no. 16 (2021): 8491, 10.3390/ijms22168491. PMC8395185 34445197

[5] R. L. Nielsen , M. B. Houlind , and A. L. Andersen , “Ponsegromab for the Treatment of Cancer Cachexia,” New England Journal of Medicine 392, no. 10 (2025): 1035–1036, 10.1056/NEJMc2500502. 40043244

[6] J. Taniguchi , S. Mikura , S. da , and K. Lopes , “The Efficacy and Safety of anamorelin for Patients with Cancer‐related Anorexia/Cachexia Syndrome: a Systematic Review and Meta‐analysis,” Scientific Reports 13, no. 1 (2023): 15257, 10.1038/s41598-023-42446-x. PMC10502008 37709824

[7] T. Setiawan , I. N. Sari , Y. T. Wijaya , et al., “Cancer Cachexia: Molecular Mechanisms and Treatment Strategies,” Journal of Hematology & Oncology 16, no. 1 (2023): 54, 10.1186/s13045-023-01454-0. PMC10204324 37217930

[8] A. Dolly , J. F. Dumas , and S. Servais , “Cancer Cachexia and Skeletal Muscle Atrophy in Clinical Studies: What Do We Really Know?,” Journal of Cachexia, Sarcopenia and Muscle 11, no. 6 (2020): 1413–1428, 10.1002/jcsm.12633. PMC7749617 33053604

[9] M. Qin , J. Zhu , L. Xing , et al., “Adipose‐derived Exosomes Ameliorate Skeletal Muscle Atrophy via miR‐146a‐5p/IGF‐1R Signaling,” Journal of Nanobiotechnology 22, no. 1 (2024): 754, 10.1186/s12951-024-02983-7. PMC11657514 39696303

[10] X. Li , X. Zhao , Z. Qin , J. Li , B. Sun , and L. Liu , “Regulation of Calcium Homeostasis in Endoplasmic Reticulum–mitochondria Crosstalk: Implications for Skeletal Muscle Atrophy,” Cell Communication and Signaling 23, no. 1 (2025): 17, 10.1186/s12964-024-02014-w. PMC11721261 39789595

[11] R. Sun , S. Zhang , W. Hu , et al., “Valproic Acid Attenuates Skeletal Muscle Wasting by Inhibiting C/EBPβ‐regulated atrogin1 Expression in Cancer Cachexia,” American Journal of Physiology‐Cell Physiology 311, no. 1 (2016): C101–C115, 10.1152/ajpcell.00344.2015. 27122162

[12] K. Xie , H. Xiong , W. Xiao , et al., “Downregulation of miR‐29c Promotes Muscle Wasting by Modulating the Activity of Leukemia Inhibitory Factor in Lung Cancer Cachexia,” Cancer Cell International 21, no. 1 (2021): 627, 10.1186/s12935-021-02332-w. PMC8626920 34838029

[13] W. Hu , Z. Ru , Y. Zhou , et al., “Lung Cancer‐derived Extracellular Vesicles Induced Myotube Atrophy and Adipocyte Lipolysis via the Extracellular IL‐6‐mediated STAT3 Pathway,” Biochimica et Biophysica Acta (BBA)—Molecular and Cell Biology of Lipids 1864, no. 8 (2019): 1091–1102, 10.1016/j.bbalip.2019.04.006. 31002945

[14] B. R. Pryce , A. Oles , E. E. Talbert , et al., “Muscle Inflammation Is Regulated by NF‐κB from Multiple Cells to Control Distinct States of Wasting in Cancer Cachexia,” Cell Reports 43, no. 11 (2024): 114925, 10.1016/j.celrep.2024.114925. PMC11774514 39475511

[15] H. Oda , A. Annibaldi , D. L. Kastner , and I. Aksentijevich , “Genetic Regulation of Cell Death: Insights from Autoinflammatory Diseases,” Annual Review of Immunology 43, no. 1 (2025): 313–342, 10.1146/annurev-immunol-090222-105848. 40279314

[16] J. Gong , X. Yao , T. Shang , X. Li , B. Chen , and H. Sun , “Molecular Mechanisms of Skeletal Muscle Atrophy: Clinical Challenges and Future Therapeutic Strategies,” Biochemical Pharmacology 249 (2026): 117913, 10.1016/j.bcp.2026.117913. 41865897

[17] J. Mallard , L. Debrut , E. Hucteau , et al., “NF‐κB Signaling Drives Rapid Skeletal Muscle Atrophy in Patients with Breast Cancer: the Cost of Fighting Cancer with Chemotherapy,” American Journal of Physiology‐Cell Physiology 330, no. 5 (2026): C1427–C1442, 10.1152/ajpcell.00148.2026. 41989455

[18] D.‐J. Fu , Y.‐J. Wu , C. Chen , C. Zhang , Z.‐Q. Su , and T. Wang , “Pevonedistat Targeting NEDDylation Activating Enzyme for human Diseases: Underlying Mechanisms, Clinical Studies, and Future Directions,” Pharmacological Research 225 (2026): 108134, 10.1016/j.phrs.2026.108134. 41687727

[19] D. Nayak , L. Jia , P. dos Santos Bury , et al., “Cryo‐EM Structures of UBA6 Reveal Mechanisms of E1–E2 Specificity and Dual FAT10/Ubiquitin Thioester Transfer,” Nature Communications 17, no. 1 (2026): 3302, 10.1038/s41467-026-69882-3. PMC13065764 41764162

[20] S. Padmakumar , A. Madhavan , B. G. Nair , and G. B. Kumar , “Pathogen‐induced Hijacking of Host SUMOylation: from Molecular Mechanisms and Prospects for Therapeutic Modulation,” Frontiers in Immunology 16 (2025): 1686880, 10.3389/fimmu.2025.1686880. PMC12702859 41403937

[21] C. Arkinson , K. C. Dong , C. L. Gee , et al., “NUB1 traps Unfolded FAT10 for Ubiquitin‐independent Degradation by the 26S Proteasome,” Nature Structural & Molecular Biology 32, no. 9 (2025): 1752–1765, 10.1038/s41594-025-01527-3. PMC12285911 40217121

[22] S. Jantrapirom , L. Lo Piccolo , and M. Yamaguchi , “Non‐Proteasomal UbL‐UbA Family of Proteins in Neurodegeneration,” International Journal of Molecular Sciences 20, no. 8 (2019): 1893, 10.3390/ijms20081893. PMC6514573 30999567

[23] Y. Y. Jeong , J. Her , and I. K. Chung , “NEDD8 ultimate Buster‐1 Regulates the Abundance of TRF1 at Telomeres by Promoting Its Proteasomal Degradation,” FEBS Letters 590, no. 12 (2016): 1776–1790, 10.1002/1873-3468.12221. 27214791

[24] N. Rani , A. Aichem , G. Schmidtke , S. G. Kreft , and M. Groettrup , “FAT10 and NUB1L Bind to the VWA Domain of Rpn10 and Rpn1 to Enable Proteasome‐mediated Proteolysis,” Nature Communications 3, no. 1 (2012): 749, 10.1038/ncomms1752. 22434192

[25] F. Brockmann , N. Catone , C. Wünsch , et al., “FAT10 and NUB1L Cooperate to Activate the 26S Proteasome,” Life Science Alliance 6, no. 8 (2023): 202201463, 10.26508/lsa.202201463. PMC10185811 37188463

[26] D. Du , W. Zhang , D. Zhang , et al., “NUB1 reduction Promotes PCNA‐mediated Tumor Growth by Disturbing the PCNA Polyubiquitination/NEDDylation in Hepatocellular Carcinoma Cells,” Cell Death & Disease 16, no. 1 (2025): 228, 10.1038/s41419-025-07567-3. PMC11958677 40164590

[27] D. Zhang , P. Wu , Z. Zhang , et al., “Overexpression of Negative Regulator of Ubiquitin‐Like Proteins 1 (NUB1) Inhibits Proliferation and Invasion of Gastric Cancer Cells through Upregulation of p27Kip1 and Inhibition of Epithelial‐mesenchymal Transition,” Pathology—Research and Practice 216, no. 8 (2020): 153002, 10.1016/j.prp.2020.153002. 32703484

[28] M. Arshad , A. R. Abdullah , F. Ismail , et al., “Complete NUB1 Depletion in ER—Negative Breast Cancer Progression in Paired Primary–metastatic Cases: a Case Series,” Journal of Medical Case Reports 20, no. 1 (2026): 230, 10.1186/s13256-026-05939-7. PMC13130408 41866577

[29] K.‐L. Tan , S. Haider , C. E. Zois , et al., “Low Cytoplasmic NUB1 Protein Exerts Hypoxic Cell Death with Poorer Prognosis in Oestrogen Receptor Negative Breast Cancer Patients,” Translational Oncology 49 (2024): 102106, 10.1016/j.tranon.2024.102106. PMC11387679 39182365

[30] M. Arshad , A. R. Abdullah , F. Ismail , et al., “Mechanism and Predictive Role of NUB1 Protein in Oestrogen Receptor Pathway of FEC‐Treated Breast Cancer Patients,” Biomedicines 13, no. 6 (2025): 1307, 10.3390/biomedicines13061307. PMC12189139 40564026

[31] B. Lu , I. Al‐Ramahi , A. Valencia , et al., “Identification of NUB1 as a Suppressor of Mutant Huntingtin Toxicity via Enhanced Protein Clearance,” Nature Neuroscience 16, no. 5 (2013): 562–570, 10.1038/nn.3367. 23525043

[32] W. Hu , H. Xiong , Z. Ru , et al., “Extracellular Vesicles‐released Parathyroid Hormone‐related Protein from Lewis Lung Carcinoma Induces Lipolysis and Adipose Tissue Browning in Cancer Cachexia,” Cell Death & Disease 12, no. 1 (2021): 134, 10.1038/s41419-020-03382-0. PMC7843996 33510128

[33] H. Xiong , J. Ye , Q. Luo , W. Li , N. Xu , and H. Yang , “Exosomal EIF5A Derived from Lewis Lung Carcinoma Induced Adipocyte Wasting in Cancer Cachexia,” Cellular Signalling 112 (2023): 110901, 10.1016/j.cellsig.2023.110901. 37743008

[34] M. D. Goncalves , S.‐K. Hwang , C. Pauli , et al., “Fenofibrate Prevents Skeletal Muscle Loss in Mice with Lung Cancer,” Proceedings of the National Academy of Sciences 115, no. 4 (2018): E743–e752, 10.1073/pnas.1714703115. PMC5789923 29311302

[35] A. L. Queiroz , E. Dantas , S. Ramsamooj , et al., “Blocking ActRIIB and Restoring Appetite Reverses Cachexia and Improves Survival in Mice with Lung Cancer,” Nature Communications 13, no. 1 (2022): 4633, 10.1038/s41467-022-32135-0. PMC9360437 35941104

[36] T. Gicquel , F. Marchiano , G. Reyes‐Castellanos , et al., “Integrative Study of Skeletal Muscle Mitochondrial Dysfunction in a Murine Pancreatic Cancer‐induced Cachexia Model,” Elife 13 (2024): RP93312, 10.7554/eLife.93312. PMC11488855 39422661

[37] G. J. Fernandez , J. H. Ferreira , I. J. Vechetti , et al., “MicroRNA‐mRNA Co‐sequencing Identifies Transcriptional and Post‐transcriptional Regulatory Networks Underlying Muscle Wasting in Cancer Cachexia,” Frontiers in Genetics 11 (2020): 541, 10.3389/fgene.2020.00541. PMC7272700 32547603

[38] V. Spadafora , B. R. Pryce , A. Oles , et al., “Optimization of a Mouse Model of Pancreatic Cancer to Simulate the human Phenotypes of Metastasis and Cachexia,” BMC Cancer 24, no. 1 (2024): 414, 10.1186/s12885-024-12104-0. PMC10993462 38570770

[39] S. N. Bilgic , A. Domaniku , B. Toledo , et al., “EDA2R–NIK Signalling Promotes Muscle Atrophy Linked to Cancer Cachexia,” Nature 617, no. 7962 (2023): 827–834, 10.1038/s41586-023-06047-y. 37165186

[40] Q. Yang , Y. Wang , C. Zhao , S. Pang , J. Lu , and P. Chan , “α‐Synuclein Aggregation Causes Muscle Atrophy through Neuromuscular Junction Degeneration,” Journal of Cachexia, Sarcopenia and Muscle 14, no. 1 (2023): 226–242, 10.1002/jcsm.13123. PMC9891985 36416282

[41] J. Farup , J. Just , F. de Paoli , et al., “Human Skeletal Muscle CD90^+^ Fibro‐adipogenic Progenitors Are Associated with Muscle Degeneration in Type 2 Diabetic Patients,” Cell Metabolism 33, no. 11 (2021): 2201–2214.e10.e2211, 10.1016/j.cmet.2021.10.001. PMC9165662 34678202

[42] V. Zoglio , S. Chebouti , F. Issa , et al., “DAXX‐dependent H3.3 Deposition Maintains Myoblast Cell Identity Independently of Other Histone Chaperone Complexes,” iScience 28, no. 8 (2025): 113119, 10.1016/j.isci.2025.113119. PMC12355108 40822342

[43] Y. Zhang , R. D. Nipp , T. Janowitz , et al., “Cancer Cachexia: a Tumor‐driven Disorder of Whole‐body Homeostasis,” Cancer Cell 44 (2026): 913–932, 10.1016/j.ccell.2026.03.012. PMC13570801 41997136

[44] S. Talebi , S. Zeraattalab‐Motlagh , M. Barkhordar , et al., “Dose‐Dependent Effect of Megestrol Acetate Supplementation in Cancer Patients with Anorexia–cachexia Syndrome: a Meta‐Analysis,” Journal of Cachexia, Sarcopenia and Muscle 15, no. 4 (2024): 1254–1263, 10.1002/jcsm.13500. PMC11294013 39031821

[45] P. Sidaway , “Palliative Care: Anamorelin Provides Benefit to Patients with Cachexia,” Nature Reviews Clinical Oncology 15, no. 2 (2018): 68, 10.1038/nrclinonc.2017.204. 29255237

[46] K. Yamamoto , Y. Kurokawa , Y. Miyazaki , et al., “Efficacy and Safety of anamorelin for Cancer Cachexia in Patients with Unresectable or Recurrent Gastric Cancer: a Multicentre, Open‐label, Randomised Controlled Trial,” EClinicalMedicine 88 (2025): 103500, 10.1016/j.eclinm.2025.103500. PMC12572812 41181834

[47] Y. Kitajima , K. Yoshioka , and N. Suzuki , “The Ubiquitin–proteasome System in Regulation of the Skeletal Muscle Homeostasis and Atrophy: from Basic Science to Disorders,” The Journal of Physiological Sciences 70, no. 1 (2020): 40, 10.1186/s12576-020-00768-9. PMC10717345 32938372

[48] H. Baazim , L. Antonio‐Herrera , and A. Bergthaler , “The Interplay of Immunology and Cachexia in Infection and Cancer,” Nature Reviews Immunology 22, no. 5 (2022): 309–321, 10.1038/s41577-021-00624-w. PMC8489366 34608281

[49] O. Rom and A. Z. Reznick , “The Role of E3 Ubiquitin‐ligases MuRF‐1 and MAFbx in Loss of Skeletal Muscle Mass,” Free Radical Biology and Medicine 98 (2016): 218–230, 10.1016/j.freeradbiomed.2015.12.031. 26738803

[50] V. K. Rao , D. Das , and R. Taneja , “Cancer Cachexia: Signaling and Transcriptional Regulation of Muscle Catabolic Genes,” Cancers 14, no. 17 (2022): 4258, 10.3390/cancers14174258. PMC9454911 36077789

[51] M. Oyabu , M. Sakaue , A. Kubo , et al., “Loss of FoxO in Skeletal Muscle Leads to Disrupted Muscle Metabolism and Exacerbates Starvation‐induced Hepatic Steatosis,” Proceedings of the National Academy of Sciences 123, no. 15 (2026): 2600036123, 10.1073/pnas.2600036123. PMC13079935 41950099

[52] G. Zhang , B. Jin , and Y. P. Li , “C/EBPβ Mediates Tumour‐Induced Ubiquitin Ligase atrogin1/MAFbx Upregulation and Muscle Wasting,” The EMBO Journal 30, no. 20 (2011): 4323–4335, 10.1038/emboj.2011.292. PMC3199382 21847090

[53] T. Kamitani , K. Kito , T. Fukuda‐Kamitani , and E. T. H. Yeh , “Targeting of NEDD8 and Its Conjugates for Proteasomal Degradation by NUB1,” Journal of Biological Chemistry 276, no. 49 (2001): 46655–46660, 10.1074/jbc.M108636200. 11585840

[54] R. Guarascio , D. Salih , M. Yasvoina , F. A. Edwards , M. E. Cheetham , and J. van der Spuy , “Negative Regulator of Ubiquitin‐Like Protein 1 Modulates the Autophagy–lysosomal Pathway via p62 to Facilitate the Extracellular Release of Tau Following Proteasome Impairment,” Human Molecular Genetics 29, no. 1 (2020): 80–96, 10.1093/hmg/ddz255. 31691796

[55] S. Sendo , C. R. L. Machado , D. L. Boyle , et al., “Dysregulated NUB1 and Neddylation Enhances Rheumatoid Arthritis Fibroblast‐like Synoviocyte Inflammatory Responses,” Arthritis & Rheumatology 76, no. 8 (2024): 1252–1262, 10.1002/art.42856. 38566346

[56] Y. Li , H. Jiang , R. Chen , H. Huang , and S. Ding , “NF‐κB Signaling as a Central Driver of Cancer Cachexia,” Cancers 18, no. 4 (2026): 557, 10.3390/cancers18040557. PMC12938688 41749811

[57] B. Zhao , G. Shi , J. Shi , et al., “Research Progress on the Mechanism and Treatment of Cachexia Based on Tumor Microenvironment,” Nutrition 133 (2025): 112697, 10.1016/j.nut.2025.112697. 39999652

[58] M. Tabebordbar , K. A. Lagerborg , A. Stanton , et al., “Directed Evolution of a family of AAV Capsid Variants Enabling Potent Muscle‐directed Gene Delivery across Species,” Cell 184, no. 19 (2021): 4919–4938.e22.e4922, 10.1016/j.cell.2021.08.028. PMC9344975 34506722

[59] J. Weinmann , S. Weis , J. Sippel , et al., “Identification of a Myotropic AAV by Massively Parallel in Vivo Evaluation of Barcoded Capsid Variants,” Nature Communications 11, no. 1 (2020): 5432, 10.1038/s41467-020-19230-w. PMC7595228 33116134

[60] S. E. van Staalduine , V. Bianco , P. Ferraro , et al., “Deciphering Structural Complexity of Brain, Joint, and Muscle Tissues Using Fourier Ptychographic Scattered Light Microscopy,” View 6 (2025): 20240143, 10.1101/2024.11.28.625428.

[61] J. Ji , E. Lipkow , N. Anton , C. Crucifix , P. Eberling , and J. Laporte , “Myomerger‐derived Peptide Enhances Skeletal Muscle Tropism and Reduces Liver Transduction of Lipid Nanoparticles for Gene Delivery,” Molecular Therapy Nucleic Acids 37, no. 1 (2026): 102785, 10.1016/j.omtn.2025.102785. PMC12741376 41458876

[62] H. Mao , X. Zhao , and S. C. Sun , “NF‐κB in Inflammation and Cancer,” Cellular & Molecular Immunology 22, no. 8 (2025): 811–839, 10.1038/s41423-025-01310-w. PMC12310982 40562870

[63] V. Ramadass , T. Vaiyapuri , and V. Tergaonkar , “Small Molecule NF‐κB Pathway Inhibitors in Clinic,” International Journal of Molecular Sciences 21, no. 14 (2020): 5164, 10.3390/ijms21145164. PMC7404026 32708302

[64] S.‐Y. Liu , L.‐K. Chen , Y.‐T. Jhong , et al., “Endothelin‐1 Impairs Skeletal Muscle Myogenesis and Development via ETB Receptors and p38 MAPK Signaling Pathway,” Clinical Science (Lond) 138, no. 12 (2024): 711–723, 10.1042/cs20240341. 38804865

[65] M. Wu , H. Li , J. He , et al., “TRIM72 Alleviates Muscle Inflammation in Mdx Mice via Promoting Mitophagy‐Mediated NLRP3 Inflammasome Inactivation,” Oxid Med Cell Longev 2023 (2023): 8408574, 10.1155/2023/8408574. PMC9876702 36713032

[66] J. Li , M. C. Chan , Y. Yu , et al., “miR‐29b Contributes to Multiple Types of Muscle Atrophy,” Nature Communications 8, no. 1 (2017): 15201, 10.1038/ncomms15201. PMC5458521 28541289

[67] Y. Wu , J. Che , P. Jia , et al., “Near‐infrared Light‐triggered Polypyrrole Promotes C2C12 Cell Differentiation and Inhibits TNF‐α Induced Myotube Atrophy,” Cellular Signalling 100 (2022): 110463, 10.1016/j.cellsig.2022.110463. 36075558

[68] F. U. R. Bhatti , K. A. Hasty , and H. Cho , “Anti‐inflammatory Role of TPCA‐1 Encapsulated Nanosomes in Porcine Chondrocytes against TNF‐α Stimulation,” Inflammopharmacology 27, no. 5 (2019): 1011–1019, 10.1007/s10787-018-0542-5. 30600473

[69] X. A. Zhu , S. Starosta , M. Ferrer , et al., “A Neuroimmune Circuit Mediates Cancer Cachexia‐associated Apathy,” Science 388, no. 6743 (2025): adm8857, 10.1126/science.adm8857. PMC13051291 40208971

[70] Y. Xia , Y. Han , W. Li , et al., “PEBP4 alleviates Muscle Wasting in Lung Cancer Cachexia via KEAP1–NRF2‐mediated Redox Homeostasis,” Cell Death & Disease 17, no. 1 (2026): 692, 10.1038/s41419-026-08925-5. PMC13443934 42236665

[71] A. Guo , K. Huang , Q. Lu , B. Tao , K. Li , and D. Jiang , “TRIM16 facilitates SIRT‐1‐Dependent Regulation of Antioxidant Response to Alleviate Age‐Related Sarcopenia,” Journal of Cachexia, Sarcopenia and Muscle 15, no. 5 (2024): 2056–2070, 10.1002/jcsm.13553. PMC11446700 39192479

[72] L. Weiss , M. Carrer , A. Shmara , et al., “Skeletal Muscle Effects of Antisense Oligonucleotides Targeting Glycogen Synthase 1 in a Mouse Model of Pompe Disease,” Clinical and Translational Medicine 15, no. 4 (2025): 70314, 10.1002/ctm2.70314. PMC12017901 40268518

